# Architectured ZnO–Cu particles for facile manufacturing of integrated Li-ion electrodes

**DOI:** 10.1038/s41598-020-69141-5

**Published:** 2020-07-24

**Authors:** Fabio L. Bargardi, Juliette Billaud, Claire Villevieille, Florian Bouville, André R. Studart

**Affiliations:** 1 Complex Materials, Department of Materials, ETH Zürich, 8093 Zurich, Switzerland; 2 Electrochemical Laboratory, Paul Scherrer Institut, 5232 Villigen, Switzerland; 3 Present Address: Centre for Advanced Structural Ceramics, Imperial College London, London, SW7 2AZ UK

**Keywords:** Batteries, Synthesis and processing

## Abstract

Designing electrodes with tailored architecture is an efficient mean to enhance the performance of metal-ion batteries by minimizing electronic and ionic transport limitations and increasing the fraction of active material in the electrode. However, the fabrication of architectured electrodes often involves multiple laborious steps that are not directly scalable to current manufacturing platforms. Here, we propose a processing route in which Cu-coated ZnO powders are directly shaped into architectured electrodes using a simple uniaxial pressing step. Uniaxial pressing leads to a percolating Cu phase with enhanced electrical conductivity between the active ZnO particles and improved mechanical stability, thus dispensing the use of carbon-based additives and polymeric binders in the electrode composition. The additive-free percolating copper network obtained upon pressing leads to highly loaded integrated anodes displaying volumetric charge capacity 6–10 fold higher than Cu-free ZnO films and that matches the electrochemical performance reported for advanced cathode structures. Achieving this high charge capacity using a readily available pressing tool makes this approach a promising route for the facile manufacturing of high-performance electrodes at large industrial scales.

## Introduction

The selection, design and manufacturing of electrode materials are key for the performance of batteries, since they directly determine the charge capacity and the rate at which the device can be charged^1–3^. To increase the charge density of Li-ion battery anodes, extensive research has been dedicated to materials exhibiting high theoretical storage capacity using alloys, conversion materials or even metallic lithium. By forming lithiated compounds with high concentrations of lithium, these materials can reach theoretical specific charges as high as 3,860 mAh/g for metallic lithium^4^ or 4,200 mAh/g for silicon^5^, which is about one order of magnitude larger than that achievable in commercial graphite anodes^6,7^. Since state-of-the-art anodes contain pores, organic binders, electrically-conductive additives and current collectors that do not store lithium, efforts have also been made to minimize the fraction of such inactive materials without impairing the functionality of the device^8–10^. The reduction of inactive materials represents a promising route to enhance the specific charge of Li-ion batteries but several additional boundary conditions still have to be considered before they become viable commercial options. Such conditions include for example high applicable charging rates, low manufacturing costs, wide availability of raw materials and long-term durability of the device under cyclic conditions^11^.

An enticing strategy that has been recently explored to fulfil these several demands is to devise novel electrode architectures, in which the spatial organization of active and inactive material is designed to maximize electrochemical performance and lift ion and electron transport limitations. As an illustrative example, electrodes with aligned porosity have been fabricated with the help of an external magnetic field to enhance by threefold the transport of Li ions across graphite anodes^12^. Similar improvements have been made by casting electrode pastes onto unidirectional sacrificial templates, followed by removal of the template by thermal treatment^13^. Anodes with hierarchical porous structure have also been developed to optimize the porosity of the electrode^14,15^. Provided that the transport of Li ions is the main factor limiting the electrochemical performance of the device, these approaches can be used to increase the volumetric charge capacity of the electrodes without compromising the achievable charging rates. To also ensure sufficient electrical conductivity of the electrode, methods have also been proposed to incorporate a metallic phase into the active material to enhance the transport of electrons beyond what is currently achieved using carbon-based additives (e.g. super-P)^16–18^. This has been achieved using for example copper, nickel and carbon-based foams, percolating networks^19,20^ or electrodeposited nanopillars^21,22^. In one of such strategies, coating the conversion material Nb_2_O_5_ with graphene resulted in anodes with reduced transport limitations, allowing for an increase of the active material loading without a severe decrease of the electrochemical performance^9^.

While the design and fabrication of architectured electrodes is clearly a very promising strategy to enhance the performance of batteries^23^, further research is still needed to enable the manufacturing of advanced electrodes with optimized microstructures and robust long-term operation using scalable, cost-effective processing technologies. Integrated electrodes made by depositing active material on pre-fabricated metallic foams show excellent electrical conductivity but are still too porous to reach high volumetric efficiency^17^. Although the architectured electrodes based on a conversion material and a percolating graphene network show a reduced detrimental effect of an increased active material loading, their fabrication involves complex steps that introduce additional processing costs and are challenging to upscale. Therefore, manufacturing technologies that can be easily translated into existing fabrication platforms and that result in electrodes with high volumetric efficiency are highly demanded. Additionally, it is important to consider all cell components when measuring the true volumetric efficiency of the cell. The active material mass often does not even reach half of the total mass of the electrode and cannot therefore be representative for the true electrochemical performance^24^.

Here, we report a manufacturing route for the fabrication of integrated electrodes with high charge capacity that is based on the room-temperature compaction of architectured particles using a simple uniaxial pressing operation. The crucial aspect of this approach is the synthesis of anode materials that are partially coated with metallic islands that will form a percolating conductive network and exert a binding function between the active material after the consolidation process. By enhancing the electrical conductivity of the anode, such metallic phase allows for the facile manufacturing of electrodes that are significantly thicker compared to copper-free counterparts. The presence of a strong percolating metal network in intimate contact with the active material also mitigates the catastrophic effect resulting from the pulverisation of the conversion material during long term cycling^25^. While the approach should be compatible with a broad range of active materials, we demonstrate the feasibility of this concept using a model conversion based material ZnO particles coated with copper islands as an illustrative example^26^.

## Results and discussion

**Figure 1:**
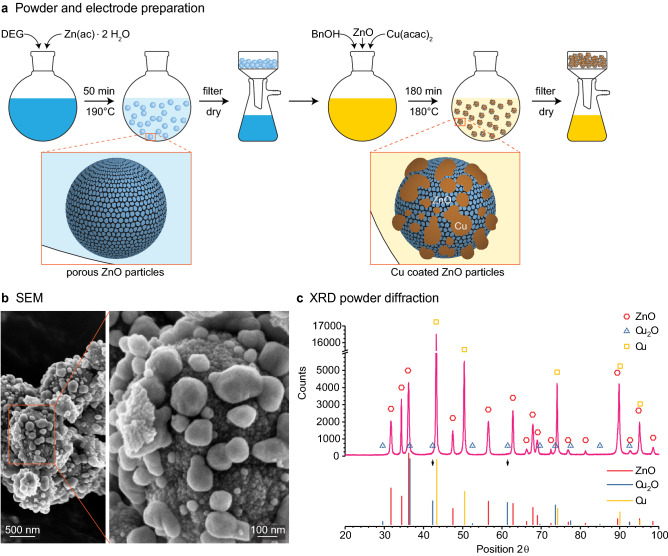
Synthesis of architectured Cu-coated ZnO particles. (**a**) Schematics of the two-step synthetic procedure based on non-aqueous sol–gel chemistry. (**b**) SEM image and (**c**) X-ray powder diffraction of the synthesized architectured particles.

The formation of architectured ZnO–Cu particles involves two simple and robust non-aqueous sol–gel reactions (Fig. 1a)^27^. In the first reaction, porous ZnO particles are prepared by mixing the Zn precursor in diethylene glycol for 50 min at 190 °C. The precipitated ZnO particles are afterwards filtered, dried and suspended in benzyl alcohol for the next step. In the second reaction, a Cu precursor is added to the ZnO suspension to form the metallic coating on the oxide particles. The coating is created while the suspension is kept under stirring at 180 °C for 180 min.

This facile synthetic route leads to Cu-coated ZnO particles architectured at submicron length scales (Fig. 1b). Scanning electron microscopy shows that the ZnO particles formed in the first step of the synthesis exhibit a hierarchical structure of 10–20 nm nanoparticles assembled into larger 400–800 nm spherical agglomerates (Fig. 1b; Supplementary Fig. S4b). In the second processing step, such agglomerates are coated with copper islands that vary in size from approximately 50 to 200 nm. The formation of metallic islands suggests that the underlying ZnO particles are not fully wetted by the precipitated copper oxide that transform into metallic copper later during the reaction. This is a desired feature for the intended electrode application, since it ensures direct exposure of the conversion material to the Li^+^ ions present in the electrolyte. Besides direct exposure to the electrolyte, the ZnO agglomerates should display high porosity to enhance the diffusion of the Li^+^ ions and accommodate the volumetric expansion resulting from the lithiation process. ZnO particles prepared under similar synthesis conditions in previous work^28^ show a total surface area of 80 m^2^/g, which is in agreement with the highly porous nature of particles produced via this non-aqueous sol–gel route, also seen in the SEM images of Fig. 1b.

XRD powder diffraction confirms that ZnO and Cu are the main phases present in the architectured powder (Fig. 1c). The absence of bulk copper oxides in the XRD spectrum indicates that the copper precursor is completely reduced into metallic copper during the second sol–gel reaction. This does not exclude the possible presence of a nanolayer of copper oxide on the surface of the bulk copper, which would not be detectable by XRD. The bulk electrical resistance of a 11 mm diameter pressed electrode was found to be less than 1 Ω (Supplementary Figure S6). This indicates that the copper particles form a conductive percolating network, which would not be expected in case the particles would be covered by a thick oxide layer. While we use here ZnO as an example of a conversion anode, the non-aqueous sol–gel reaction could be applied to a variety of oxides and should thus be applicable to other electrode materials (see Supplementary Figure. S2). In addition to its chemical versatility, this synthetic procedure also allows for easy tuning of the metallic coverage by changing the amount of reactants used in the synthesis (see Supplementary Fig. S3).

**Figure 2:**
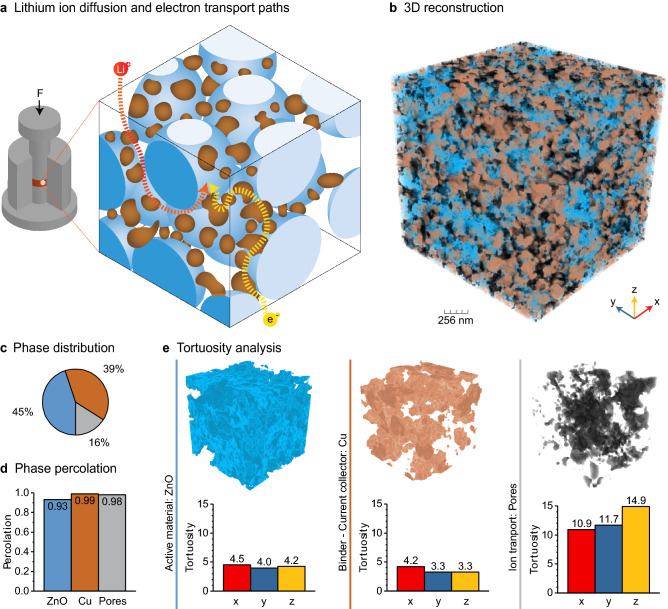
Microstructure of the integrated electrodes by uniaxial pressing of the powder. (**a**) Schematics of the tri-continuous interpenetrating network of ZnO, Cu and pore phases obtained upon pressing of the architectured powder. (**b**) 3D reconstruction of the interpenetrating phases obtained from FIB cross sections of a pressed electrode. (**c**) Volume fraction and (**d**) percolation degree of the ZnO, Cu and porous phases across the electrode. (**e**) Calculated tortuosity of the individual phases along specific directions within the structure.

Architectured Cu-coated ZnO particles allow for the facile manufacturing of integrated electrodes by uniaxial pressing of the powder in the dry state (Fig. 2a). The resulting integrated electrode combines into one single bulk part: (1) the electrochemically active conversion material (ZnO), (2) a porous network for the diffusion of Li^+^ ions through the electrolyte, and (3) a metallic network for the transport and collection of electrons ensuring also the mechanical stability of the electrode. Combined these features allowed us to obtain an integrated electrode architecture with high volumetric charge.

To fully benefit from this integrated architecture in a battery electrode, it is essential that the porous and metallic network show high degree of percolation across the entire structure. We assessed the structure and the degree of percolation of the integrated electrode by analysing reconstructed images obtained from 3D FIB tomography of a specimen pressed at 160 MPa (Fig. 2b). Our results show that the pressing step increases the apparent density of the electrode, leading to a monolith comprising ca. 45 vol% of mesoporous ZnO, ca. 39 vol% of Cu and ca. 16 vol% of macropores (Fig. 2c). Comparison of the volume fraction of phases obtained from the FIB data with the relative density measured using the Archimedes method suggests that the ZnO phase contains ca. 18% mesoporosity. This indicates that the electrodes reached a relative density of 66% after pressing. The image analysis also reveals that all these phases form a tri-continuous interpenetrating network, each of which with a percolation degree higher than 0.93 (Fig. 2d). The formation of a percolating metallic phase indicates that the pressure applied during compaction and the concentration of copper in the architectured powder were sufficiently high to establish contact points between the metallic islands present on the surface of the ZnO particles. The high relative density of the electrode after pressing (ca. 66% or up to ca. 84% without counting the ZnO mesoporosity) also suggests that the copper islands probably underwent localized plastic deformation during compaction of the powder, providing a simple way to produce high-density electrodes at room temperature.

While percolation ensures the formation of a continuous path for the transport of electrons and Li ions across the structure, the tortuosity of the percolating network determines the resistance of the electrode against these electronic and ionic transport processes. By solving numerically the diffusion equation to estimate the mass transport in 3D for each one of the phases, we obtain tortuosity values in the ranges of 4.0–4.5, 3.2–4.2 and 10.9–14.9 for the ZnO, Cu and porous phases, respectively. The higher tortuosity of the porous network reflects the significantly lower volume fraction of macropores relative to the other phases (Fig. 2e). The tortuosity of the porous phase is slightly higher along the vertical (z) direction, most likely due to the structural anisotropy generated during uniaxial pressing. Given the strong dependence of the tortuosity on the volume fraction of the respective phase^2^, this important structural parameter can be potentially tuned by adjusting the relative fraction of the ZnO and Cu in the initial powder and the pressure applied in the manufacturing process.

**Figure 3:**
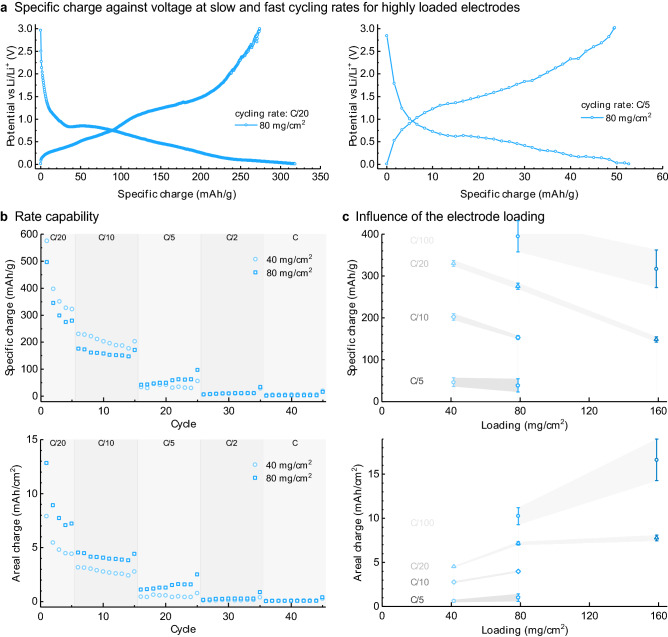
Electrochemical performance of the pressed Cu–ZnO anodes. (**a**) Representative galvanostatic curves for electrodes loaded with 80 mg/cm^2^ of powder for different cycling rate (C/20 and C/5) between 0.01 and 3 V vs. Li ^+^/Li. (**b,c**) Effect of (**b**) the applied cycling rate on electrodes with different loading and (**c**) the electrode loading on the charge capability of samples pressed at 160 MPa and exhibiting total porosity of approximately 44%.

The effect of this percolating architecture on the electrochemical performance of the electrode was evaluated by performing galvanostatic measurements and rate capability tests on specimens with fixed powder loading. The electrochemical behaviour of the architectured anode is first assessed by examining the lithiation process of the ZnO particles under the low charging rate of C/20. This allows us to determine the different Li-based compounds formed during conversion of the ZnO to the lithiated products. Indeed, the galvanostatic data show that the potential of the electrode displays distinct plateaus when plotted as a function of the specific charge introduced in the material (Fig. 3a). The shape of the potential curves below 1.5 V is characteristic of ZnO under lithiation. The four potential plateaus at 1.3 V, 0.7 V, 0.5 V and 0.4 V can be assigned to various Li–Zn phases^29,30^. We cannot exclude that some features below 1.5 V might also be associated to reactions between Li^+^ and Cu/Cu_2_O^31^. This feature is not observed in conventional electrodes deposited on copper current collectors since those current collectors have a preferential orientation limiting their reaction with Li^+^. The sloping region above 2 V is attributed to the electrochemical activity of copper, since ZnO does not store charge at such high potential. As opposed to conventional flat current collectors, the Cu phase within our electrode architecture exhibits a much higher surface area, thus increasing the apparent electrochemical activity of Cu. We estimate the amount of charge stored in the Cu phase to be around 1/3 of the total charge, which corresponds to approximately 125 mAh/g for all cycling rate tested here, based on electrochemical tests performed on copper coated alumina electrodes (see discussion in the SI and Supplementary Fig S2). This storage capacity of Cu is similar to that observed with carbon additives and graphene scaffolds. For instance, a graphene oxide scaffold can reach values between 190 and 210 mAh/g at 100 mA/g and 2,400 mA/g, respectively^32^. Taking into account that hand-made (laboratory) electrodes may contain up to 20 wt% of this additive, a non-negligible amount of their specific charge arises from the charging capacity of the carbon present in the electrode formulation^33^.

Increasing the charging rate from C/20 to C/5 significantly changes the profile of the potential curves (Fig. 3b). At C/5 rate, the electrochemical load curve is smooth and the potential plateaus are no longer clearly observed. Moreover, a clear overpotential is visible, due to, among others, an increased resistance of the system towards the diffusion of Li^+^ in the electrolyte compared to the tests performed at C/20 rate. To better understand the electrochemical behaviour of the architectured anode under such diffusion-limited conditions, we performed galvanostatic rate capability tests while keeping the powder loading fixed at 40 mg/cm^2^ (Fig. 3b). The experimental results show the expected reduction of the specific charge of the electrode as the charging rate is increased. Despite the observed diffusion limitations observed at high charge rates, the specific charge of our highly loaded electrode at C/10 reaches 200 mAh/g, which is 1.5–2 times higher than the values reported for thin ZnO electrodes containing 20 wt% of additives and tested at comparable charging rates^29,34^. Although inferior than the specific charge of 490 mAh/g obtained with ZnO–Ni foams at C/12.5 rate^34^, our electrodes can be readily prepared in a simple pressing operation and thus do not require the multi-step processes required for the manufacturing of the foam-based anode.

The ability to prepare thick electrodes is another major advantage of our anodes, since this increases the fraction of active material in the battery, possibly enhancing the attainable specific charge per unit volume of the total device. Too thick electrodes, however, increase the diffusion length for electrons and Li^+^ ions, which in turn reduces the rate at which the battery can be charged and discharged. We examined the effect of the electrode thickness on the electrochemical response of the anodes by measuring the specific charge of specimens prepared with powder loadings varying from 40 to 160 mg/cm^2^ (Fig. 3c). Such powder loadings led to high electrode thickness in the range of 100–400 µm. Our experimental data confirms that the charging rate needed to reach a specific charge per unit mass of the electrodes decreases for increasing powder loadings. This is probably related to the low porosity of the pressed anodes, which limits the diffusion of Li^+^ ions throughout the electrode structure.

**Figure 4:**
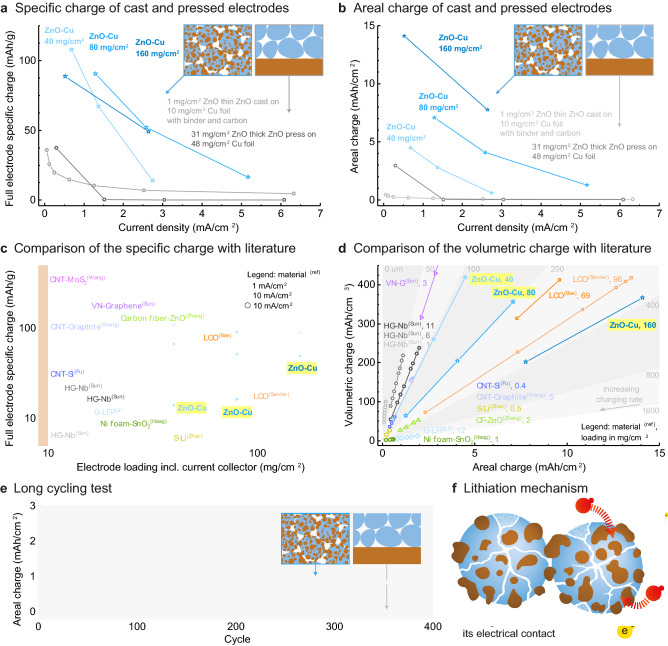
Electrochemical performance of the pressed Cu–ZnO anodes compared to conventionally-processed ZnO anodes and various architectured electrodes reported in the literature. (**a,b**) Comparison with cast and pressed ZnO electrodes in terms of (**a**) specific charge and (**b**) areal charge. (**c**) Specific charge as a function of powder loading for a wide range of architectured anode and cathode materials. (**d**) Estimated area and volumetric charges of the Cu–ZnO architectured electrodes compared to the literature. (**e**) Long-term cycling test of a Cu–ZnO electrode conducted at C/10 rate as compared to the performance of a reference specimen of ZnO deposited on a copper foil with the same ZnO and Cu amounts. (**f**) Lithiation and electron transport is still possible after particle fracture due to the binding role of the copper phase.

Despite their diffusion limitations, the proposed integrated anodes perform very well if compared with reference ZnO specimens that do not feature a percolating metallic network (Fig. 4a). To quantify the performance improvement arising from the presence of a percolating metal phase, we compare the specific charge of the architectured electrodes with those of reference anodes prepared by pressing or slip-casting of Cu-free ZnO powders. Given the capacitive effect of the metallic phase, the specific charge was calculated considering the mass of both ZnO and Cu phases. For the cast specimens, the ZnO powder was suspended in a solvent containing 20 wt% of poly(vinylidene difluoride) as binder and 20 wt% of a carbon additive (Super P). The resulting suspension was directly cast onto a Cu foil to create a thin film with particle loading of 1.5 mg/cm^2^ (corresponding to 0.9 mg/cm^2^ ZnO). In the case of the pressed reference samples, a thick anode with loading of 31 mg/cm^2^ was prepared by uniaxially pressing ZnO powder onto a 48 mg/cm^2^ Cu foil. The electrochemical data obtained from these comparative measurements indicate that the metallic network enhances the specific charge of the anode by 4–5 times when compared to the reference specimens at current densities in the range 1–3 mA/cm^2^ (Fig. 4a). The relative improvement in capacity reaches 1 order of magnitude if the electrochemical performance of the electrode is quantified in terms of areal charge (Fig. 4b). Although the difference in specific charge becomes smaller at higher current densities, the remarkable effect of the metallic percolating network is clearly demonstrated and can possibly be maintained at high current densities if the diffusion of Li^+^ at high rates is facilitated through the incorporation of aligned porosity across the anode, as demonstrated by previous work^12,13, 22^.

When compared with other Li-ion electrodes reported in the literature, our architectured Cu–ZnO anodes stand out in terms of powder loading (Fig. 4c). While the stronger diffusion limitations associated with these high powder loadings inevitably reduces the specific charge that can be stored per unit gram of anode material, the high estimated areal and volumetric charge capacity is a key advantage of such highly-loaded electrodes (Fig. 4d). With a volumetric charge of up to 400 mAh/cm^3^ at 5 mA/g current density, the Cu–ZnO anodes surpass the volume-based capacity of several previously-reported integrated electrodes, including the Ni foam-SnO_2_, graphene-Nb_2_O_5_ and graphite systems^9,35,36^. Indeed, the specific charges achieved are comparable to those of high-capacity architectured cathodes, with which they can be potentially combined to create high-performance full cells^3,13^. Moreover, the high performance of the architectured anodes can be achieved using uniaxial pressing as a very simple manufacturing procedure. This contrasts with the multi-step labor-intensive processes needed to fabricate the high-performance electrodes used as references^3,13,37^. Reducing the manufacturing process to such a straightforward operation is only possible due to the unique structure of the initial Cu-coated ZnO powder.

The metallic percolating network obtained by pressing this powder not only incorporates current collecting capabilities to the electrodes but also enables long-term cyclic operation of the conversion material used for Li^+^ uptake. This is demonstrated by cyclic tests performed at C/10 rate on Cu–ZnO specimens with powder loading of 80 mg/cm^2^ (Fig. 4e). The cyclic measurement reveals that the electrode sample retains 80% of its initial charge for 35 cycles and is able to operate for as many as 400 cycles while keeping an areal charge of 0.8 mAh/cm^2^, which corresponds to 31% of the charge capacity shown in the beginning of the test. By contrast, a reference sample comprising a ZnO film deposited on a Cu foil, therefore same composition but different architecture, cannot withstand more than 3 cycles at 10 mA/g current density before losing its charging capabilities. The high cycling performance of the integrated Cu–ZnO electrode is quite impressive in view of the large volumetric expansion in the order of 200% expected during lithiation of this conversion material^38^. For comparison, conversion anodes exhibiting similar volumetric changes often display very limited cycling capabilities^39^. The high stability of the Cu–ZnO anodes against cycling results from the interconnecting role of the Cu phase in between the ZnO particles (Supplementary Fig. S5). The large number of contact points between the active material and the copper provides mechanical integrity to the anode even after the expected fragmentation of the ZnO particles during lithiation (Fig. 4e). The presence of the binding Cu phase ensures that electrical contact is still maintained throughout the electrode. Our results suggest that even after fracture of the particles upon cycling the conductivity is retained due to the copper coating as schematically illustrated in Fig. 4f.

## Conclusions

Thick integrated electrodes featuring high volumetric charge capacity can be easily manufactured by uniaxial pressing of architectured Cu-coated ZnO particles. The high volumetric charge of the electrodes arises from the high relative density of the electrode and the absence of organic binders and carbon-based additives in the anode structure. The fabrication of functional electrodes with high loading is possible through the formation of a three-dimensional percolating network of copper upon pressing of the initial architectured powder. This metallic network provides electrical conductivity and mechanical stability to the thick electrode during cycling. With an areal charge of 14 mAh/cm^2^ and a volumetric capacity of up to 400 mAh/cm^3^ at a current density of 5 mA/cm^2^, the pressed Cu–ZnO electrodes can store 6 times more charge as compared to conventional ZnO films deposited on Cu foils and perform comparably well with the most advanced architectured electrodes reported in the literature. Moreover, the high charge density of the pressed anodes is above 80% capacity retention for 35 cycles and degrades by 69% when the electrode is subjected to 400 charging cycles, an impressive performance for the active material used here. This long-term cycling capability contrasts with the quick failure of the reference anode. This is possible due to the binding role of the copper network, which prevents full fragmentation of the active material during the volumetric changes associated with the lithiation process. Most importantly, such electrochemical behaviour is achieved using a straightforward manufacturing process that is simple enough to be entirely compatible with the existing workflow of today’s battery production. The synthetic procedure used to obtain the architectured powder can in principle be applied to a wide range of other conversion materials that suffer from the same cycling issues. By programming the electrode architecture already during the synthesis of the anode material, this approach enables the utilization of a readily available pressing operation for the scalable manufacturing of electrodes with enhanced electrochemical performance.

## Methods

### Chemicals

Zinc acetate dihydrate [Zn(ac)_2_.2H_2_O, 99–101%] was purchased from Sigma Aldrich. Copper(II) acetylacetonate [Cu(acac)_2_, 98%], benzylalcohol (BnOH, 99%) and diethylene glycol (DEG, 99%, extra pure) were purchased from Acros. *N*-Methyl-2-pyrrolidone (NMP, 99%) was purchased from Fluka. The Super P carbon black and the poly(vinylidene difluoride) binder (PVDF, Kynar) were purchased from Imerys (Switzerland) and Arkema, respectively. All chemicals were used as received. The Li metal counter electrode (≥ 99.9%, 0.75 mm thickness), the glass fibre separator and the LP30 electrolyte were purchased from Alfa Aesar, Whatman UK and BASF, respectively. Such an electrolyte contains 1 M LiPF_6_ dissolved in a 1:1 weight ratio of ethylene carbonate and dimethyl carbonate.

### Synthesis of ZnO nanoparticles

Nanoparticles were prepared by adapting a procedure published elsewhere^27, 40^. In brief, 40 g of Zn(ac) were added to 200 mL DEG in a round bottom flask and immersed in a preheated oil bath at 190 °C. As soon as the temperature of the solvent reached 154 °C, the powder fully dissolved, and the solution became transparent. When the solution reached a temperature of 170 °C, it became turbid, a sign of the nucleation of the ZnO nanoparticles. After 30 min, the reaction was stopped, the flask was removed from the oil bath, and the solution let to cool down to ambient temperature. The suspended product was collected by filtering, washed with 100 mL of ethanol, and dried under vacuum at 1 mbar for 24 h.

### Copper coating of ZnO nanoparticles

1 g of ZnO nanoparticles were first dispersed in 300 mL of BnOH and tip sonicated for 15 min at 100 W under magnetic stirring. Then, 8.55 g of Cu(acac)_2_ were added and the resulting suspension was immersed in a pre-heated oil bath at 180 °C for 3 h 45 min under continuous stirring. The flask was allowed to cool down to room temperature and the powder was separated from the solvent by vacuum filtration and washed with 100 mL of ethanol. The powder was then dried in a vacuum of 1 mbar for 24 h. The yield of this synthesis is 98%.

### Powder characterization

Powder samples were sputtered with 3 nm of platinum and investigated by scanning electron microscopy (SEM, LEO 1530, Zeiss, Germany). X-ray diffraction (XRD) measurements of the powders were performed at room temperature with an XRD diffractometer (X’Pert Pro, PANalytical) for 2θ angles between 20° and 100° for 35 min operating in reflection mode with Cu Kα radiation (45 kV, 40 mA).

### Preparation of pressed electrodes

20, 40 and 80 mg of the coated powder were placed in an 8 mm diameter pressing mould (Specac, Switzerland) and pressures ranging from 40 MPa (2 kN) to 640 MPa (32 kN) were applied using a mechanical testing machine (Instron 8562, USA) to prepare mechanically stable electrodes. The powder loadings varied from 40 to 160 mg/cm^2^.

### Preparation of tape cast electrodes

83.3 mg of PVDF was dissolved in 2.6 mL NMP with the help of a high shear mixer (IKA Ultraturrax, Germany). Then, 83.3 mg Super P carbon black was added and mixed, followed by the incorporation and mixing of 250 mg of the active material powder into the resulting suspension. The casting was performed on a 12.5 µm thick copper foil using a custom-made tape casting device. The thickness of the wet cast layer was set to 250 µm. The dry electrode sheet showed a solid loading of 2.5 mg/cm^2^, composed of 20 wt% PVDF (binder), 20 wt% Super P carbon black and 60 wt% of the synthesized powder, leading to a total weight fraction of 21% ZnO and 39% Cu. The ZnO loading is therefore 0.5 mg/cm^2^ for the copper-coated powder electrodes. For the reference electrodes without copper coating, the solid loading was 2.2 mg/cm^2^, composed of 20 wt% binder, 20 wt% Super P carbon black and 60 wt% of ZnO powder leading to a ZnO loading of 1.3 mg/cm^2^.

### Microstructure characterization through image analysis

The microstructure of the samples was analysed via scanning electron microscopy (LEO 1530, Zeiss, Germany). The tomography data were collected on a FIB machine (NVision 40, Zeiss, Germany) and aligned with *Image J*’s plugin *Correct 3D drift*^41^. The *Matlab* script *TauFactor*^42, 43^ (version 1.208) was used to measure the phase fraction, percolation, and tortuosity. The percolation degree is a value between 0 and 1 and represents the fraction of material of the considered phase which is interconnected. The free software *Paraview* was used to produce the 3D images. Further details about the analysis are reported in Supplementary Fig. S1.

### Density determination

The density of the electrodes was calculated geometrically through the known diameter of 8 mm and the measured thickness. The theoretical density is calculated considering the volumetric fraction of each phase and assuming that densities are additive: ${d}_{th} = {d}_{ZnO}\cdot f + {d}_{Cu}\cdot \left(1-f\right)$, where *d*_*ZnO*_ = 5.61 g/cm^3^, *d*_*Cu*_ = 8.96 g/cm^3^ and *f* is the volume fraction of ZnO relative to the total volume. The relative density of the electrode was calculated using the ratio: *d*_*rel*_ = *d*/*d*_*th*_ with *d*_*th*_ = 7.504 g/cm^3^.

### Electrochemical characterization

The electrochemical performance of the electrodes was assessed on a half-cell configuration. Cast and pressed electrodes were placed in a customized coin cell with a Li metal counter electrode and a glass fibre separator (Whatman, UK). 500 μL of LP30 (BASF, Germany), containing 1 M LiPF_6_ dissolved in a 1:1 weight ratio of ethylene carbonate and dimethyl carbonate, was chosen as electrolyte. Cycling was performed in galvanostatic mode using an Astrol device (Astrol Electronic AG, Switzerland). All measurements were performed at 25 °C and all potentials cited in the text are given with respect to a Li^+^/Li reference. In a typical testing program 5 cycles at C/20 are first applied to enable the formation of the solid electrolyte interphase (SEI). This is followed by 10 cycles at C/10, C/5, C/2, C and 2C rates. Finally, 400 cycles at C/10 rate are conducted to test the long-term stability of the electrode. For a 1C-rate, a current of 1,000 mAh/g is applied to (dis-) charge the battery in 1 h. The cell is cycled between the cut-off potentials of 0.01 and 3 V. A potentiostatic step of one hour was performed at 3 V before each change in the cycling rate to allow the electrode to reach an equilibrium and to assess the real electrochemical performance of the electrode. Since the copper phase also stores electrical charge, the specific charge obtained from the electrochemical measurements was normalized by the total electrode mass. To compare the volumetric specific charge of our integrated electrodes to that of reference anodes and cathodes reported in the literature, we assume the current collector of the reference anodes to show a minimal mass of 10 mg/cm^2^ and a thickness of 12.5 µm. Our integrated electrodes do not need an additional current collector because of the intrinsic conductivity properties of the 3D copper network.

## Supplementary information

Supplementary information

## Data Availability

The data that support the finding of this study are available from the corresponding author on request.

## References

[1] Lukatskaya MR, Dunn B, Gogotsi Y (2016). Multidimensional materials and device architectures for future hybrid energy storage. Nat. Commun..

[2] Ebner M, Chung DW, García RE, Wood V (2014). Tortuosity anisotropy in lithium-ion battery electrodes. Adv. Energy Mater..

[3] Bae C-J, Erdonmez CK, Halloran JW, Chiang Y-M (2013). Design of battery electrodes with dual-scale porosity to minimize tortuosity and maximize performance. Adv. Mater..

[4] Zhang Y (2017). High-capacity, low-tortuosity, and channel-guided lithium metal anode. Proc. Natl. Acad. Sci..

[5] Bruce PG, Scrosati B, Tarascon J-M (2008). Nanomaterials for rechargeable lithium batteries. Angew. Chem. Int. Ed..

[6] Tarascon JM, Armand M (2001). Issues and challenges facing rechargeable lithium batteries. Nature.

[7] Buqa H, Goers D, Holzapfel M, Spahr ME, Novák P (2005). High rate capability of graphite negative electrodes for lithium-ion batteries. J. Electrochem. Soc..

[8] Bibienne T (2017). Eco-friendly process toward collector- and binder-free, high-energy density electrodes for lithium-ion batteries. J. Solid State Electrochem..

[9] Sun H (2017). Three-dimensional holey-graphene/niobia composite architectures for ultrahigh-rate energy storage. Science (80-).

[10] Wang B (2013). High volumetric capacity silicon-based lithium battery anodes by nanoscale system engineering. Nano Lett..

[11] Gallagher KG (2016). Optimizing areal capacities through understanding the limitations of lithium-ion electrodes. J. Electrochem. Soc..

[12] Billaud J, Bouville F, Magrini T, Villevieille C, Studart AR (2016). Magnetically aligned graphite electrodes for high-rate performance Li-ion batteries. Nat. Energy.

[13] Sander JS, Erb RM, Li L, Gurijala A, Chiang Y-M (2016). High-performance battery electrodes via magnetic templating. Nat. Energy.

[14] Minas C (2018). Freezing of gelled suspensions: A facile route toward mesoporous TiO_2_ particles for high-capacity lithium-ion electrodes. ACS Appl. Nano Mater..

[15] Xie D, Zhang M, Wu Y, Xiang L, Tang Y (2020). A flexible dual-ion battery based on sodium-ion quasi-solid-state electrolyte with long cycling life. Adv. Funct. Mater..

[16] Wang L, Han J, Kong D, Tao Y, Yang Q-H (2019). Enhanced roles of carbon architectures in high-performance lithium-ion batteries. Nano-Micro Lett..

[17] Jin S, Jiang Y, Ji H, Yu Y (2018). Advanced 3D current collectors for lithium-based batteries. Adv. Mater..

[18] Wu D, Zhang W, Feng Y, Ma J (2020). Necklace-like carbon nanofibers encapsulating V 3 S 4 microspheres for ultrafast and stable potassium-ion storage. J. Mater. Chem. A.

[19] Zhao Q (2015). Sulfur nanodots electrodeposited on Ni foam as high-performance cathode for Li-S batteries. Nano Lett..

[20] Ji H (2012). Ultrathin graphite foam: A three-dimensional conductive network for battery electrodes. Nano Lett..

[21] Villevieille C (2008). The good reactivity of lithium with nanostructured copper phosphide. J. Mater. Chem..

[22] Taberna PL, Mitra S, Poizot P, Simon P, Tarascon J-M (2006). High rate capabilities Fe_3_O_4_-based Cu nano-architectured electrodes for lithium-ion battery applications. Nat. Mater..

[23] Sun H (2019). Hierarchical 3D electrodes for electrochemical energy storage. Nat. Rev. Mater..

[24] Freunberger SA (2017). True performance metrics in beyond-intercalation batteries. Nat. Energy.

[25] Zhou X (2020). Strategies towards low-cost dual-ion batteries with high performance. Angew. Chemie Int. Ed..

[26] Li F (2013). Hydrothermal self-assembly of hierarchical flower-like ZnO nanospheres with nanosheets and their application in Li-ion batteries. J. Alloys Compd..

[27] Kränzlin N, Niederberger M (2013). Wet-chemical preparation of copper foam monoliths with tunable densities and complex macroscopic shapes. Adv. Mater..

[28] Jézéquel D, Guenot J, Jouini N, Fiévet F (1995). Submicrometer zinc oxide particles: Elaboration in polyol medium and morphological characteristics. J. Mater. Res..

[29] Pelliccione CJ, Ding Y, Timofeeva EV, Segre CU (2015). In situ XAFS study of the capacity fading mechanisms in ZnO anodes for lithium-ion batteries. J. Electrochem. Soc..

[30] Wang H, Pan Q, Cheng Y, Zhao J, Yin G (2009). Evaluation of ZnO nanorod arrays with dandelion-like morphology as negative electrodes for lithium-ion batteries. Electrochim. Acta.

[31] Rehnlund D (2015). Electrochemical fabrication and characterization of Cu/Cu_2_O multi-layered micro and nanorods in Li-ion batteries. Nanoscale.

[32] David L, Bhandavat R, Barrera U, Singh G (2016). Silicon oxycarbide glass-graphene composite paper electrode for long-cycle lithium-ion batteries. Nat. Commun..

[33] Gnanamuthu R, Lee CW (2011). Electrochemical properties of Super P carbon black as an anode active material for lithium-ion batteries. Mater. Chem. Phys..

[34] Zhang CQ (2007). Electrochemical performances of Ni-coated ZnO as an anode material for lithium-ion batteries. J. Electrochem. Soc..

[35] Haag JM, Pattanaik G, Durstock MF (2013). Nanostructured 3D electrode architectures for high-rate Li-ion batteries. Adv. Mater..

[36] Wang K (2013). Super-aligned carbon nanotube films as current collectors for lightweight and flexible lithium ion batteries. Adv. Funct. Mater..

[37] Fu K (2013). Aligned carbon nanotube-silicon sheets: A novel nano-architecture for flexible lithium ion battery electrodes. Adv. Mater..

[38] Li H (2016). Synthesis and electrochemical investigation of highly dispersed ZnO nanoparticles as anode material for lithium-ion batteries. Ionics (Kiel)..

[39] Cabana J, Monconduit L, Larcher D, Palacín MR (2010). Beyond intercalation-based Li-ion batteries: The state of the art and challenges of electrode materials reacting through conversion reactions. Adv. Mater..

[40] Kränzlin N, Ellenbroek S, Durán-Martín D, Niederberger M (2012). Liquid-phase deposition of freestanding copper foils and supported copper thin films and their structuring into conducting line patterns. Angew. Chem. Int. Ed..

[41] Parslow A, Cardona A, Bryson-Richardson RJ (2014). Sample drift correction following 4D confocal time-lapse imaging. J. Vis. Exp..

[42] Cooper SJ, Bertei A, Finegan DP, Brandon NP (2017). Simulated impedance of diffusion in porous media. Electrochim. Acta.

[43] Cooper SJ, Bertei A, Shearing PR, Kilner JA, Brandon NP (2016). TauFactor: An open-source application for calculating tortuosity factors from tomographic data. SoftwareX.

