# Publisher Correction: Architectured ZnO–Cu particles for facile manufacturing of integrated Li-ion electrodes

**DOI:** 10.1038/s41598-020-73842-2

**Published:** 2020-10-02

**Authors:** Fabio L. Bargardi, Juliette Billaud, Claire Villevieille, Florian Bouville, André R. Studart

**Affiliations:** 1 Complex Materials, Department of Materials, ETH Zürich, 8093 Zürich, Switzerland; 2 Electrochemical Laboratory, Paul Scherrer Institut, 5232 Villigen, Switzerland; 3 Present Address: Centre for Advanced Structural Ceramics, Imperial College London, London, SW7 2AZ UK

Correction to: *Scientific Reports* 10.1038/s41598-020-69141-5, published online 24 July 2020

This Article contains errors in Figure 4 due to a technical issue. In panel (c) the axes and markers for each material are omitted, in panel (e) the graph data for the long-term cycling test is omitted, and in panel (f) the labels are incomplete. The correct Figure 4 appears below as Figure 1.

**Figure 1:**
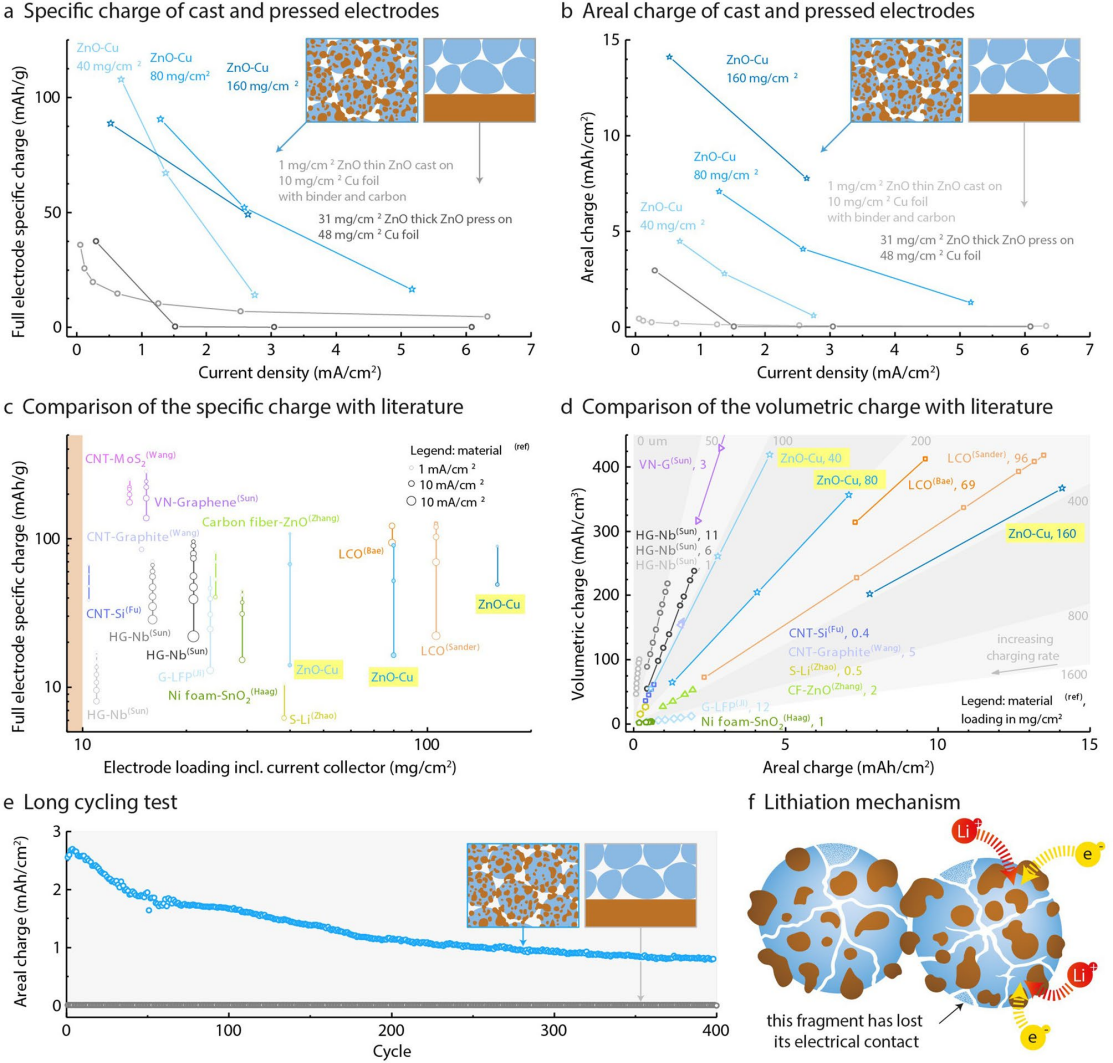
.

