# Supplementary material for: Architectured ZnO–Cu particles for facile manufacturing of integrated Li-ion electrodes
Source: Sci Rep. 2020 Jul 24;10:12401. doi: 10.1038/s41598-020-69141-5 (PMC7382461; doi:10.1038/s41598-020-69141-5)
Supplement: Supplementary file 1 — Supplementary information [file 41598_2020_69141_MOESM1_ESM.docx]

# Supplementary Information for

# Architectured ZnO-Cu particles for facile manufacturing of integrated Li-ion electrodes

## Fabio L. Bargardi^1^, Juliette Billaud^2*^, Claire Villevieille^2^, Florian Bouville^1+*^ and André R. Studart^1*^

^1^ Complex Materials, Department of Materials, ETH Zürich, CH-8093 Zürich, Switzerland

^2^ Electrochemical Laboratory, Paul Scherrer Institut, CH-5232 Villigen PSI, Switzerland

^+^: Now at Centre for Advanced Structural Ceramics, Imperial College London, SW7 2AZ London, United Kingdom

* Correspondence to: [billaud.juliette@gmail.com](mailto:billaud.juliette@gmail.com), [f.bouville@imperial.ac.uk](mailto:f.bouville@imperial.ac.uk), [andre.studart@mat.ethz.ch](mailto:andre.studart@mat.ethz.ch)

## Supplementary text

FIB/SEM analysis of Cu-ZnO electrodes. The volume fractions of Cu, ZnO and macropores in the architectured electrodes were determined from FIB slices based on the grey intensity of individual pixels (Fig. S1a). Image analyses of volumes of different sizes were performed in order to ensure that the examined volume is representative of the entire structure (Fig. S1b). The minimum representative volume obtained from this evaluation was used to quantify the percolation degree of the distinct phases in the electrode (Fig. 2, main text).

Fabrication and characterization of copper-coated α-alumina powder. Copper-coated alumina particles were prepared as a model system to estimate the contribution of the copper phase on the electrochemical performance of the ZnO-Cu electrodes. This model system was prepared by dispersing 0.33 g of α-Al_2_O_3_ particles (Ceralox, particle size <500 nm) in 100 mL of BnOH. Then, 2.85 g Cu(acac)_2_ were added to the suspension, which was immersed in a pre-heated oil bath at 180 °C for 3 h 45 min. The electrodes were fabricated by casting a slurry of 83.3 mg of PVDF, 83.3 mg of Super P carbon black and 250 mg of the copper-coated α-Al_2_O_3_ particles in 3 mL NMP.

Our electrochemical tests show that the copper phase is able to store electrical charge that amounts to 100-150 mAh/g (Fig. S2a,b). Such copper phase is mainly of metallic nature, as confirmed by the X-ray diffraction pattern of the copper-coated alumina particles (Fig. S2c). Elemental analysis suggests that the copper phase is well distributed on top and between the alumina particles (Fig. S2d,e).

Synthesis of copper-coated zinc oxide particles with different copper coverages. In addition to the particles containing 67 wt % Cu discussed in the main text, ZnO particles with varying copper concentrations were also prepared to illustrate the wide compositional range that can be covered using the non-aqueous sol-gel chemistry used in this work. Distinct copper concentrations are possible by changing the relative ratio of copper acetylacetonate relative to ZnO in the initial reaction mixture, as indicated in Table S1. X-ray diffraction of the obtained powders confirm that the fraction of Cu/ZnO can be deliberately changed using this simple approach (Fig. S3a). SEM pictures of the powders are shown in Fig. S3b. Electrochemical characterization of specimens with different Cu/ZnO ratios indicate that the specific charge of the electrode with 67 wt% of copper is 1.5 and 3 times higher compared to electrodes containing 49 wt% and 26 wt% of copper, respectively (Fig. S3c).

**Pressed electrode characterization.** The tap density is given by the mass per unit volume of the electrode at zero stress and is equal to 1.47 g/cm^3^, which corresponds to 18% relative density. The density increases up to around 5 g/cm^3^ (65% relative density) during compaction of the ZnO-Cu powder under a pressure of 400 MPa (Fig. S4a). A high magnification SEM picture of the pure ZnO powder (Fig. S4b) and a cross section of the pressed ZnO-Cu electrode (Fig. S5) reveal the microstructure of the starting conversion material and the manufactured electrode after pressing, respectively**.** Finally, the bulk electrical resistance of a 11 mm diameter electrode pressed at 160 MPa is shown to be lower than 1 Ohm (Fig. S6).


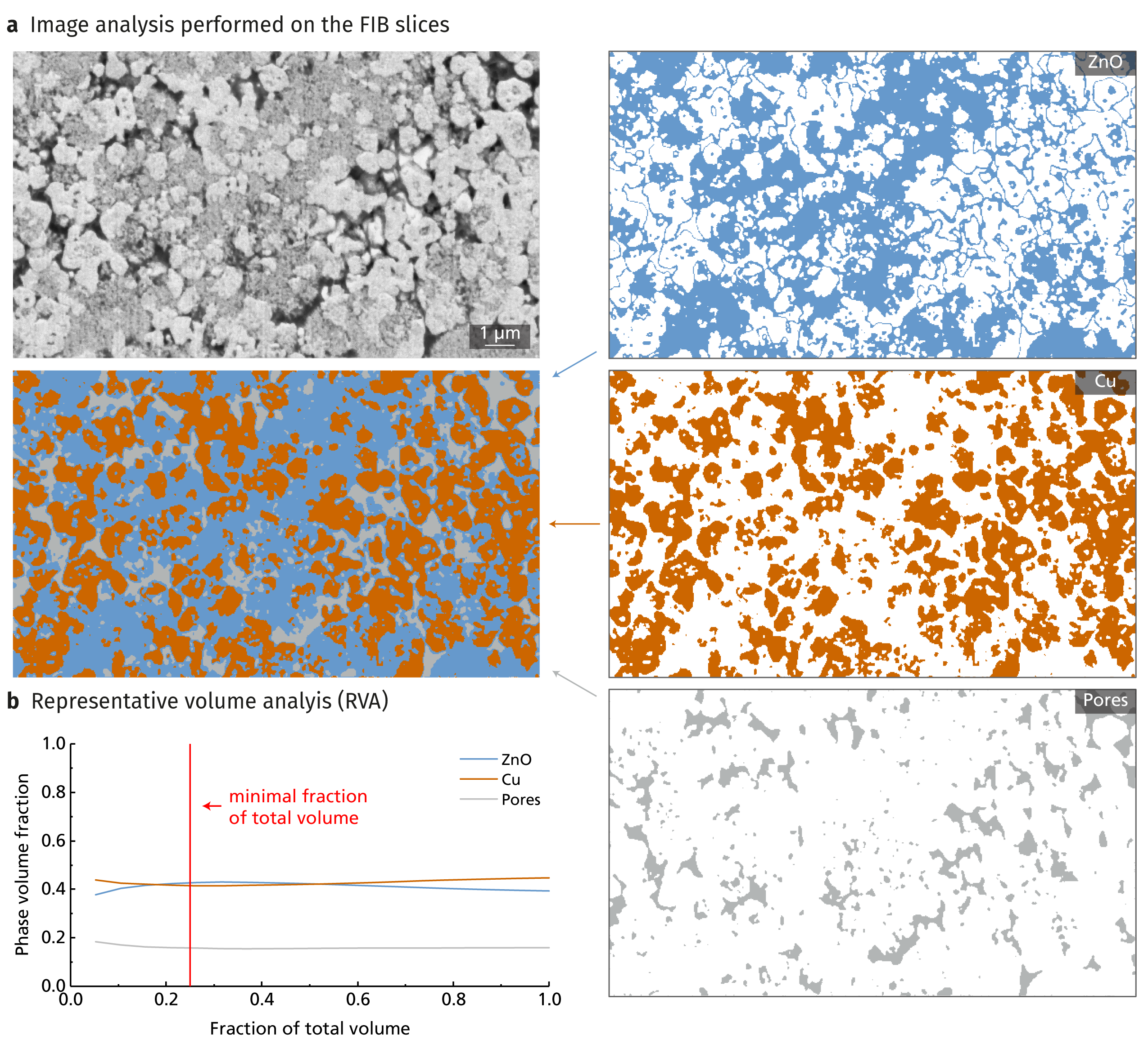


Fig. S1. FIB/SEM image analysis. (a) The phases of a FIB/SEM slice are separated based on the brightness of every single pixel (bright is copper, dark are pores and grey is ZnO). (b) The representative volume analysis (RVA) based on the relative density of each phase as a function of the fraction of the total volume imaged indicates that considering 25% of the total volume is the minimum to obtain scale independent values.


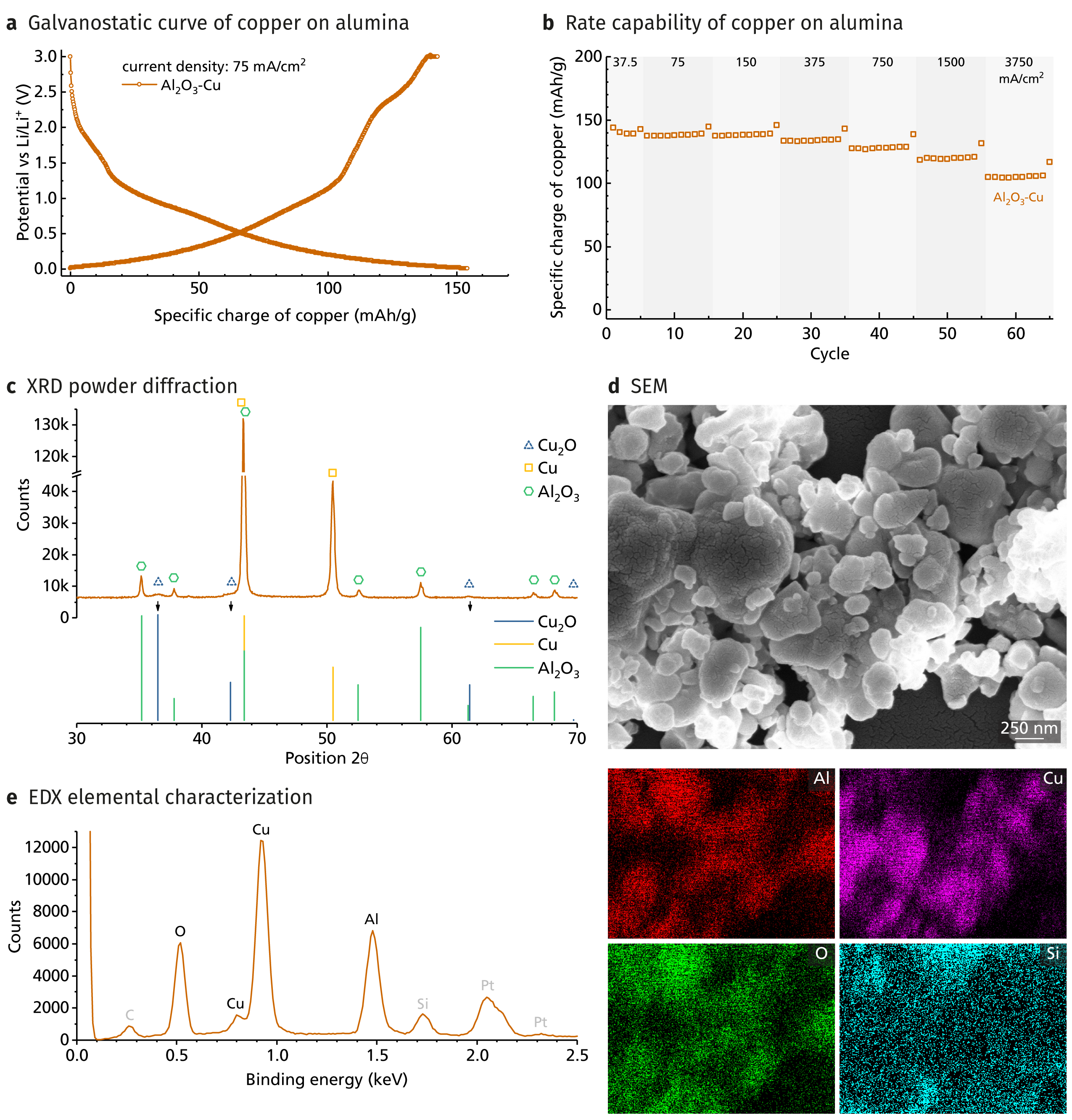


Fig. S2. Electrochemical activity and charge storage capacity of copper coated α-alumina particles. (a) Galvanostatic curves for α-Al_2_O_3_ coated with copper electrodes for a current density of 75 mA/cm^2^ between 0.01 and 3 V vs. Li^+^/Li. (b) Effect of the applied current density. (c) XRD powder diffraction. (d) SEM image and (e) EDX elemental characterization of the copper coated α-Al_2_O_3_ particles with special distribution maps for the elements Al, Cu, O, with Si originating from the glass substrate and Pt from the conductive coating layer.

**
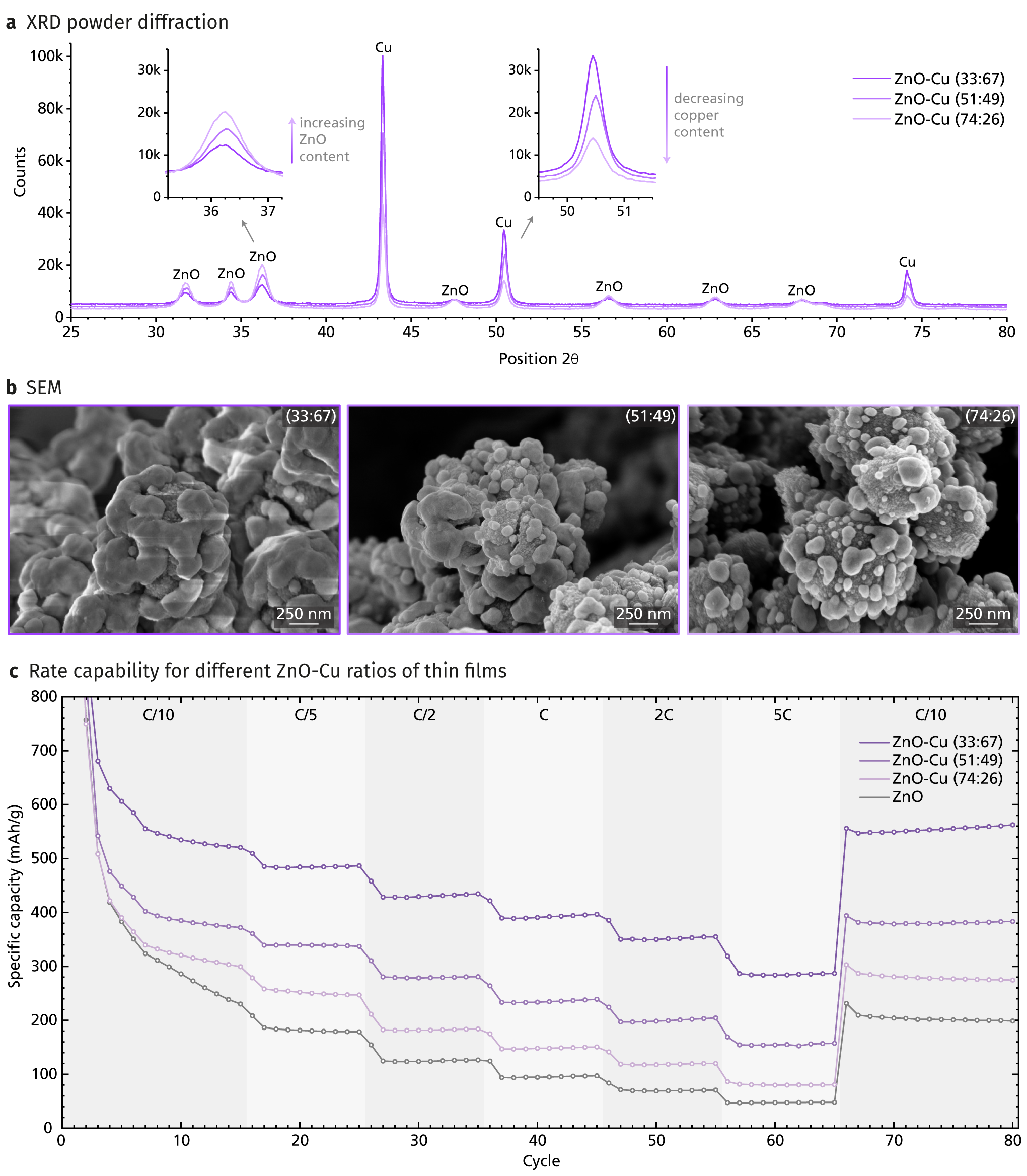
**

Fig. S3. Synthesis with different copper coating concentrations. (a) XRD powder diffraction of three powders with different copper coating concentrations. The values in brackets indicate the ratio in wt % of ZnO and Cu. Details of the synthesis parameters are reported in Table S1. (b) SEM images of the three powders. (**c**) Electrochemical performance of electrodes with different copper contents. The samples were prepared by tape-casting and contained 60 wt% of synthesized material (ZnO-Cu), 20 wt% of carbon black and 20 wt% of PVDF binder, leading to an active material loading of 1-2 mg/cm^2^.

**
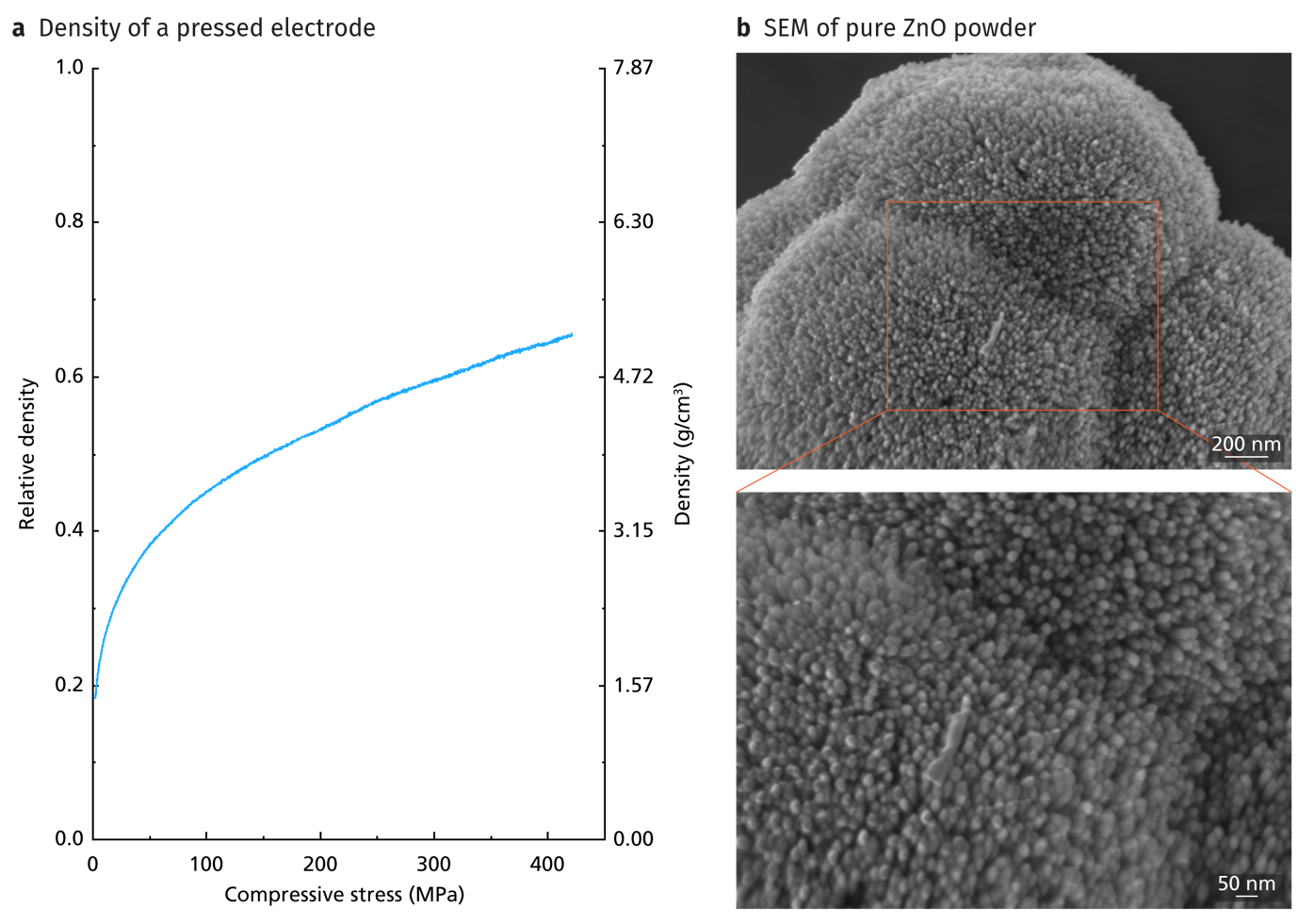
**

**Fig. S4. Powder characterization.** (a) Density increase during the compression of powder for electrode preparation, (b) SEM picture of pure ZnO after synthesis exhibiting the hierarchical structure of 10-20 nm nanoparticles assembled in larger agglomerates.


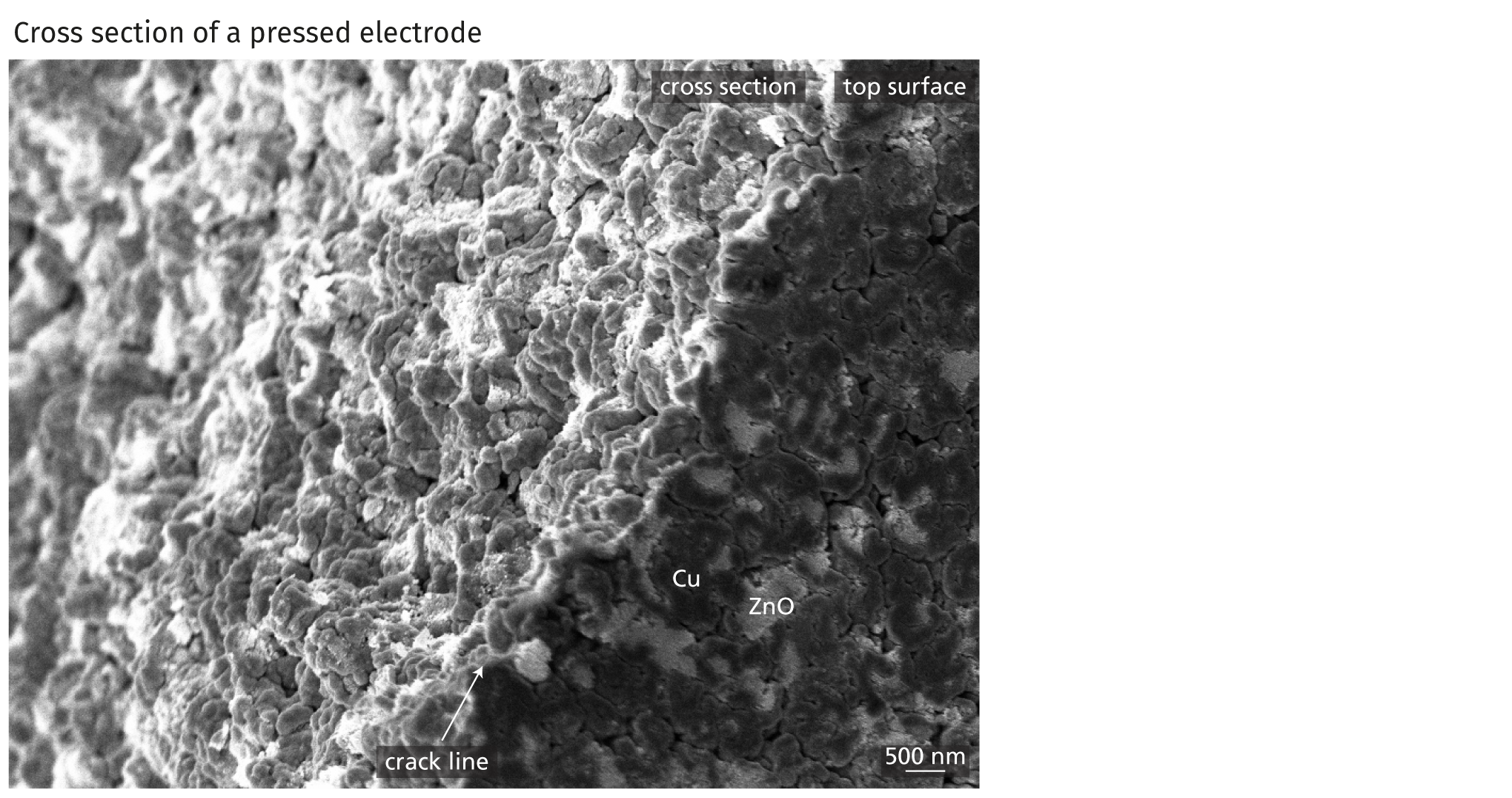


**Fig. S5. Cross section of a pressed electrode**. The fractured surface and the top surface that was in contact with the pressing tool are visible. ZnO has a lighter grey colour, whereas Cu appears in darker grey.

**
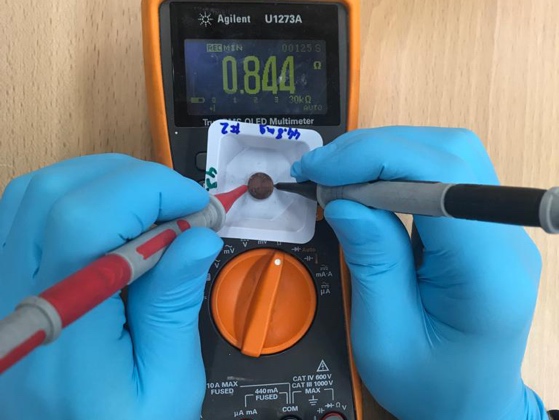
**

**Fig. S6. Bulk electrical resistance measurement.** Bulk electrical resistance measurement (in Ohms) of a 11 mm diameter electrode pressed at 160 MPa measured with a multimeter.

Table S1. Synthesis parameters for different copper coating concentrations.

|  | ZnO-Cu (33:67) | ZnO-Cu (51:49) | ZnO-Cu (74:26) |
| --- | --- | --- | --- |
| Mass of ZnO | 0.33 g | 0.66 g | 1.33 g |
| Mass of Cu(acac)_2_ | 2.85 g | 2.85 g | 2.85 g |
| Volume of BnOH | 100 mL | 100 mL | 100 mL |
| Synthesis yield | 98% | 95% | 89% |
| Final composition  (by mass calculations) | 33 wt % ZnO  67 wt % Cu | 51 wt % ZnO  49 wt % Cu | 74 wt % ZnO  26 wt % Cu |
